# A novel laboratory simulation system to uncover the mechanisms of uranium upward transport in a desert landscape

**DOI:** 10.1016/j.mex.2019.11.031

**Published:** 2019-12-03

**Authors:** Qinku Zhang, Steven L. Larson, John H. Ballard, Pohlee Cheah, Joseph A. Kazery, Heather M. Knotek-Smith, Fengxiang X. Han

**Affiliations:** a Department of Chemistry and Biochemistry, Jackson State University, 1400 J. R. Lynch Street, Jackson, MS, 39217, USA; b School of Civil Engineering and Shaanxi Key Laboratory of Ecological Restoration in Shanbei Mining Area, Yulin University, Yulin, Shaanxi, 719000, China; c U.S. Army Engineer Research and Development Center, 3909 Halls Ferry Rd., Vicksburg, MS, 39180-6199, USA

**Keywords:** A Novel Laboratory Simulation System to Uncover the Mechanisms of Uranium Upward Transport in a Desert Landscape, Contaminant, Uranium, Transport, Soil, Fraction

## Abstract

After depleted uranium (DU) is deposited in the environment, it corrodes producing mobile uranium species. The upward transport mechanism in a desert landscape is associated with the dissolution/precipitation of uranium minerals that vary in composition and solubility in soil pore water. The objective of this study is to develop the laboratory column simulation to investigate the upward transport mechanism with cyclic capillary wetting and drying moisture regimes. Results showed that evaporation driven upward transport occurred even during the first 2 months of wetting-drying regimes. Evaporation driven upward transport may control the U movement in the soil profile in an arid climate. The new system did not generate any uranium-containing wastewater.

• Simulates the upward transport process of pollutants with different pollution levels and species.
• Simultaneously simulate the transport process of multiple pollutants simultaneously.
• Evaluate the influence of biogeochemical factors on pollutant transport such as various cations and anions (Ca, Mg and carbonates) in water.

## Graphical abstract

**Figure:**
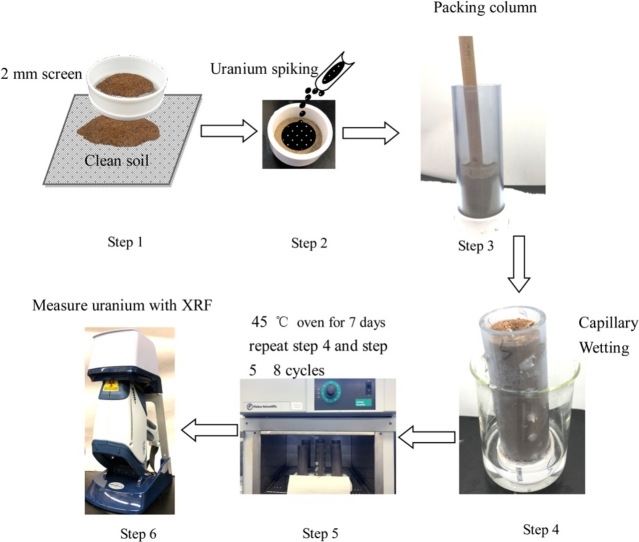

**Specification Table**

**Table:** 

Subject Area:	Environmental Science
More specific subject area:	Heavy metal pollution and control
Method name:	A Novel Laboratory Simulation System to Uncover the Mechanisms of Uranium Upward Transport in a Desert Landscape
Name and reference of original method:	F.X. Han, W.L. Kingery, J.E. Hargreaves, T.W. Walker, Effects of land uses on solid-phase distribution of micronutrients in selected vertisols of the Mississippi River Delta, Geoder, 142(2007) 96–103. [6] J. Liu, C.S. Zhao, G.Y Yuan, Y. Dong, J.J. Yang, F.Z. Li, J.L Liao, Y.Y. Yang, N. Liu, Adsorption of U (VI) on a chitosan/polyaniline composite in the presence of Ca/Mg-U (VI)-CO_3_ complexes, Hydrometallurgy, 175 (2018) 300-311. [9]
Resource availability:	This uranium metal were purchased from United Nuclear Scientific (USA). We use XRF (Bruker S1 TITAN) to measure it.

## Method details

### Background

DU is a unique metal (U + Ti alloy) that is used for both military and civil applications that require extremely high density such as kinetic energy penetrators and ballasts. These uses include ammunition armor counterweights drilling equipment, surrogate for fissionable high density materials. DU penetrators are used in weapons system for its self-sharpening, high density and hardness characteristics as a result DU alloy particulates has become distributed in some locations. During the 1991 Gulf War about 321 tons [1] of DU were fired and approximately 170–1700 tons [2] were used in the second Gulf war (2003). DU penetrators have been fired at military testing and training facilities in various countries. Uranium can be a toxic and radioactive contaminant in the environment [3], and an enhanced understanding of mobility of this metal in soils is required in order to support the sustainable use of this material.

When the DU metal is deposited in soil, processes of corrosion and transport occur as the metal allow weathers. Visual observations of autunite group minerals have been made on military testing ranges through its definitive bright yellow coloration on the soil surface and within the soil lying atop buried penetrators. The corrosion and upward transport mechanism over multiple dissolution/precipitation events of U in arid soil had not been studied in controlled conditions. A unique laboratory apparatus was required in order to perform controlled studies of these processes.

**Fig. 1:**
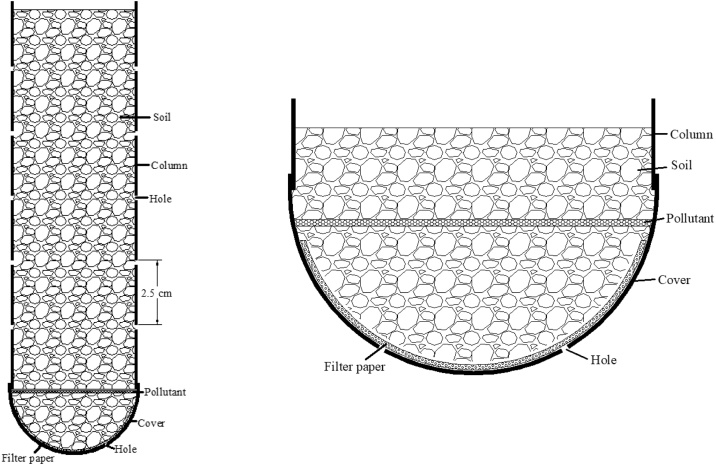
The structure diagram of the column.

The transport of U in soil was affected by various biogeochemical conditions and environmental factors, such as the initial uranium solubility of the corrosion product, the pH values of soil, the soluble ions present in the soil pore water, the mass of organic matter in soil, sizes of soil particles and soil moisture regimes etc. Column experiments were used to simulate the transport of U and other heavy metals in soil, often downward transport with gravity driven of surface/ground water. However, both gravity driven downward transport as well as evaporation driven upward transport influences the U movement through the soil profile. This novel laboratory design of a column system is to simulate capillary-evaporation driven upward transport of U in soil (Fig. 1). This system generated no uranium-containing wastewater.

### Procedures

1 Dry clean soil in the drying oven
2 Grind dry soil using ground plate and grinding rod
3 Obtain <2 mm fraction using 2 mm screen
4 Grind the desired mass of soil metal compounds (UO_2_, UO_3_, UO_2_(NO_3_)_2_) into powder with agate mortar
5 Drill a series of holes with a diameter of 0.5 cm along the column (D×H = 4 cm × 15 cm) from the bottom to the top with the interspace of 2.5 cm between holes
6 Drill the 3 mm-scale holes in the bottom cover and put one filter paper on the top of the bottom cover
7 Place a small layer of soil above the filter paper of the bottom cover
8 Weigh the proper amount of solid metal compounds and uniformly spread them on the top surface of the soil
9 Connect the column to the bottom section with the bottom cover, filter paper, a thin layer soil and a thin layer of U metal compounds
10 Add soil to the column in the heights of 5 cm with packing-packing force (slide hammer 20−25 newtons/cm^2^)
11 Place the bottom of the packed column inside the solutions containing water with the desired companion cations/anions in aqueous solution [4,5]. The position of U contaminant inside the column should be higher than the water level in order to the upward transport of U compound dissolved in pore water through capillary force when evaporation occurs
12 Place column with wet soil in oven. The temperature of the oven can be set in order to simulate temperature ranges of interest. In the current configuration the temperature was set to 45℃. The temperature simulated the hot summer in Yuma, AZ desert.
13 Repeat the wetting and drying cycles (wetting-drying, Step 12–13) when the soil of the column was completely dry (about 7 days later)
14 After repeating “wetting-drying” cycles for 2 months, remove a sample soil from the lowest hole of the column, use X-ray fluorescence spectrometer (XRF) to measure the uranium concentration in soil
15 Monitor the upward migration of uranium by sampling from the holes at different soil column heights
16 The “wetting-drying” cycles and experiments can discontinued be stopped when the U is detected at the desired study height
17 Take the soil out of the column in one piece
18 Cut the soil column into 2.5 cm layers and take 2 g mixed soil as a subsample
19 Use X-ray power diffraction (XRD) to study the U compounds in the soil
20 Run Scanning Electron Microscopy (SEM) and Energy Dispersive X-ray Spectroscopy (EDS) to study the morphology and elemental composition of samples to determine element distribution in the soil
21 Measure the fractionation of uranium in each section with the method of selective sequential dissolution (SSD) [6]
22 Calculate U transport rate in the soil under wetting-drying cycle moistures

### Final remarks

This work presents a novel laboratory uranium upward transport simulation system that provides a capability to evaluates pore water chemistries into the capillary rise uranium transport phenomenon. The procedure consisted of three steps: soil column preparation, operation of U transport driven through soil capillary and evaporation under wetting-drying cycle moistures, and analysis and characterization of stratified soil column. The traditional method required a large amount of uranium-containing solution and a diaphragm pump to feed the water continuously. The traditional column transport system is not adequate to study the mechanism of U upward transport in an arid desert landscape. This method offered systematic “wetting-drying” of the soil moistures, which better simulates the actual upward transport process and mineralogical forms of uranium in natural soil driven by evaporation. This method had the advantages of low energy consumption, low waste production and better representation of uranium transport in a natural arid environment.

## Procedures for the total uranium and fractionation of uranium in the soil samples

### Total uranium

1 The total U concentrations were detected and monitored in soils, which was taken out from small holes along the column with X-ray fluorescence spectrometer (Bruker).
2 After the end of the experiments, total U in soils were also measured with Inductively coupled plasma mass spectrometry (ICP-MS) after acid digestion.
3 For acid digestion-ICP-MS, weight 1.0 g soil sample into digesting tube (50 mL) with replicates
4 Digest the soil with 25 mL of 4 M HNO_3_ in a water-bath at 80℃ for 16 h [7,8]
5 The supernatant was decanted and filtered through a 0.45-μm filter
6 Dilute the digested soil solution
7 Measure the metal concentrations in the diluted solution with ICP-MS

### Fraction of uranium

U in soils was fractionated with the selective sequential dissolution (SSD) [6].

## Verifying the validity

**Fig. 2:**
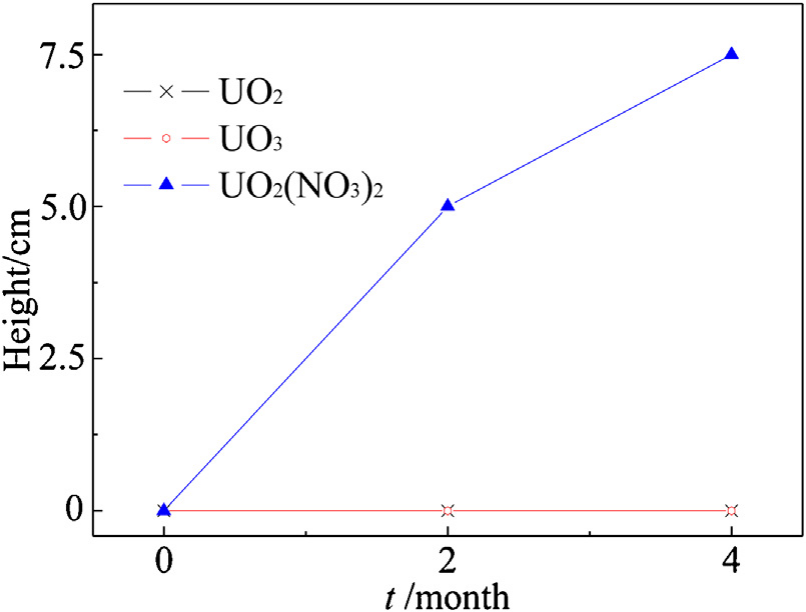
The transport of different containment in H_2_O.

Uranium compounds (UO_2_, UO_3_, UO_2_(NO_3_)_2_) were placed at the bottom of the column. Total uranium concentration in soils of the column by height was measured with XRF after “wetting-drying” cycles periodically (Fig. 2). There was no uranium detected above the initial level in the columns constructed using UO_2_ and UO_3_. The transport distance of uranium reached up to 7.5 cm after 4 months in the column of UO_2_(NO_3_)_2_. There was no significant difference (*p* = 0.05 level) in the concentration of uranium at 2.5 cm and 5 cm height after 2 and 4 months.

**Fig. 3:**
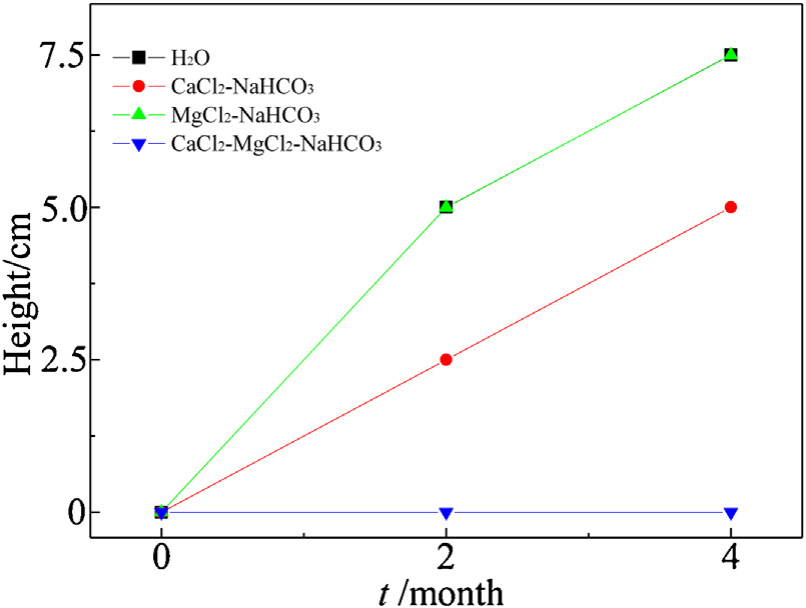
Transport of UO_2_(NO_3_)_2_ in different aqueous solutions containing CaCl_2_-NaHCO_3_, MgCl_2_-NaHCO_3_ and CaCl_2_-MgCl_2_-NaHCO_3_ solution.

To illustrate the usefulness of the method for simulations varied soil types and conditions, UO_2_(NO_3_)_2_ was placed at the bottom of the column which were contacted with aqueous solution containing different cations (tap water, 1 mmol/L CaCl_2_-NaHCO_3_ solution, 1 mmol/L MgCl_2_-NaHCO_3_ solution or 1 mmol/L CaCl_2_-MgCl_2_-NaHCO_3_ solution). These aqueous solutions were used as capillarity solution to study the effect of different cations/anions on U upward transport. Total uranium concentration was measured with XRF after “wetting-drying” cycles for every 2 months (Fig. 3). After 4 months, the transport distances of uranium in the MgCl_2_-NaHCO_3_ and CaCl_2_-NaHCO_3_ column were 7.5 cm and 5 cm in height, respectively. However, uranium was undetected in the CaCl_2_-MgCl_2_-NaHCO_3_ column. For the column of MgCl_2_-NaHCO_3_ and CaCl_2_-NaHCO_3_, no significant difference (*p* = 0.05 level) was observed in the concentration of uranium in soil at 2.5 cm and 5 cm height after 2 and 4 months. Liu et al. [9] used chitosan/polyaniline as solid-phase extractant for adsorbing U from bicarbonate-buffer solution containing Ca^2+^ and Mg^2+^, and found that the adsorption affinity was in the sequence of U-CO_3_^2−^ > Ca-U-CO_3_^2-^>Mg-U-CO_3_^2-^. Meanwhile, some similar phenomenon occurred in previous research reports [[10], [11], [12]]. This could be caused by the complexes of Ca(UO_2_)(CO_3_)_3_^2-^ and Ca_2_(UO_2_)(CO_3_)_3_ which tends to be adsorbed on the soil in neutral to weak alkaline solution.

### Conclusion

The present method offers a systematic “wetting-drying” of the soil moisture through evaporation driven capillary movement in the arid environment. This system better simulates the actual upward transport process and mineralogical forms of uranium in natural soil in a desert climate. The traditional column method often uses a peristaltic pump to feed water, requires high energy consumption and generates a large amount of contaminated wastewater. The present method has the advantages of low energy consumption, low U wastewater production, and better similarity to the uranium transport in the natural arid environment.
